# Progressive Medicine, Vol. III

**Published:** 1901-11

**Authors:** 


Progressive Medicine, Vol. III. A Quarterly Digest of Advances, Discoveries and Improvements in the Medical and Surgical Sciences. Edited by Hobart Amory Hare, M. D., Professor of Therapeutics and Materia Medica in the Jefferson Medical College of Philadelphia. Octavo, handsomely bound in cloth, 428 pages, 16 illustrations. Per annum, in four cloth bound volumes, $10. Lea Brothers & Co., Philadelphia and New York.

The contributors to this volume are: Dr. William Ewart, on the Diseases of the Thorax and its Viscera; Dr. William S. Gottheil, on Dermatology and Syphilis; Dr. William G. Spiller, on Diseases of the Nervous System; and Dr. Richard C. Norris, on Obstetrics.

Dr. Ewart presents the most recent views on the treatment of diseases of the respiratory system, including surgical treatment in all the diseases of the pleura and the lungs where such is indicated. He also pays due attention to the diagnosis and treatment of all the important diseases of the heart and blood vessels. His section is the largest, and perhaps the most interesting from the standpoint of the general practitioner.

Dr. Spiller devotes over one hundred pages to a discussion of the nervous system. He pays particular attention to cerebral tumors and abscesses.

The section on obstetrics, by Dr. Norris, contains a record of all that has been added to our knowledge in that branch during the past year. R.

